# Congenital anterolateral tibial bowing and polydactyly: a case report

**DOI:** 10.1186/1752-1947-1-54

**Published:** 2007-07-23

**Authors:** Edmond G Lemire

**Affiliations:** 1 Division of Medical Genetics, Department of Pediatrics, University of Saskatchewan, Saskatoon, SK, S7N 0W8, Canada

## Abstract

Congenital anterolateral bowing of the tibia is a rare deformity that may lead to pseudarthrosis and risk of fracture. This is commonly associated with neurofibromatosis type 1. In this report, we describe a 15-month old male with congenital anterolateral bowing of the right tibia and associated hallux duplication. This is a distinct entity with a generally favourable prognosis that should not be confused with other conditions such as neurofibromatosis type 1. Previously published cases are reviewed.

## Background

Neurofibromatosis type 1 (NF1) is a common genetic disease with a prevalence of 1 in 3000 individuals in the general population [1]. With NF1 being a relatively common condition, it is not unusual to consider it in the differential diagnosis when a case of anterolateral tibial bowing is identified in a child as this is an associated feature that can progress to pseudarthrosis and a high risk of fracture [1,2]. However bowing of the tibia is not pathognomonic for NF1. Another uncommon condition, congenital anterolateral tibial bowing and polydactyly (CATBP) presents in this manner [3]. CATBP is readily distinguishable from NF1 because of the absence of any neurocutaneous signs and the presence of hallux duplication and associated hand malformations.

In this report, we describe a 15-month old boy with CATBP who was unnecessarily investigated because of concerns regarding NF1. This entity needs to be recognized by pediatricians to avoid unnecessary investigations for a neurocutaneous disorder.

## Case report

**Figure 1:**
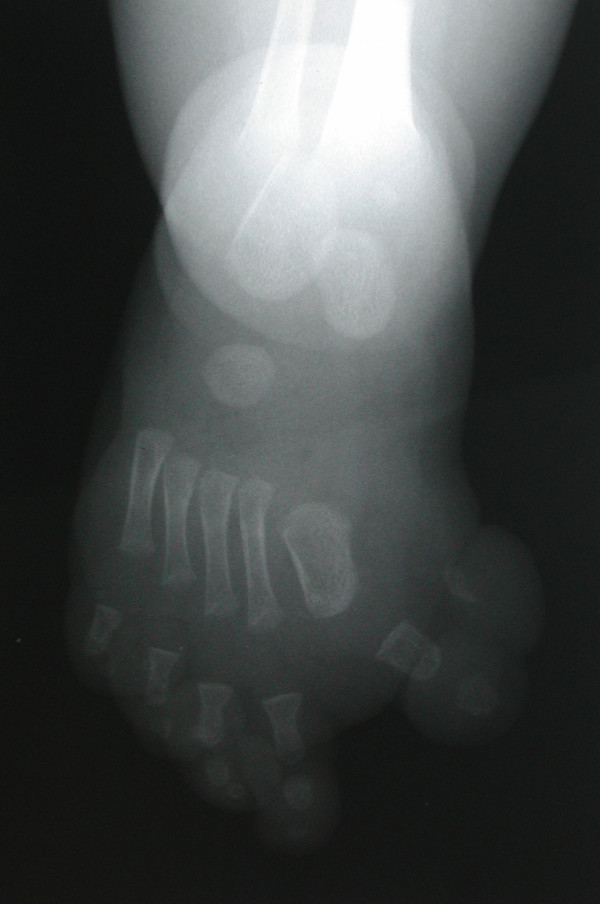
Medially directed duplicated hallux.

The patient was born at term by spontaneous vaginal delivery following induction. His growth parameters were all within normal limits for gestational age. It was observed that he had a right lower limb deformity consisting of medial splaying and duplication of the hallux with mild anterolateral bowing of the ipsilateral tibia [Figure 1]. Incidental note was made of radial deviation of the left index finger. The remainder of the physical examination was unremarkable. Specifically, there were no cutaneous manifestations suggestive of NF1 and other neurocutaneous disorders. Radiographs of the leg showed duplication of the first ray on the right. The right tibia was bowed anterolaterally and a medullary cyst was present. No other radiographic abnormalities were noted. The newborn period was otherwise unremarkable and he was discharged from hospital on the third day.

The pregnancy had been remarkable for an elevated alpha-fetoprotein level (2.86 multiples of the median (MoM)) as part of the maternal serum screen drawn in the 18^th ^week of gestation. The levels of the two other analytes were within normal limits (unconjugated estriol 1.43 MoM; human chorionic gonadotrophin 1.21 MoM). The mother's risk for an open neural tube defect was increased to 1/117 from the general population level of 1/1000. Serial antenatal ultrasounds were performed without identification of any fetal anomalies.

**Figure 2:**
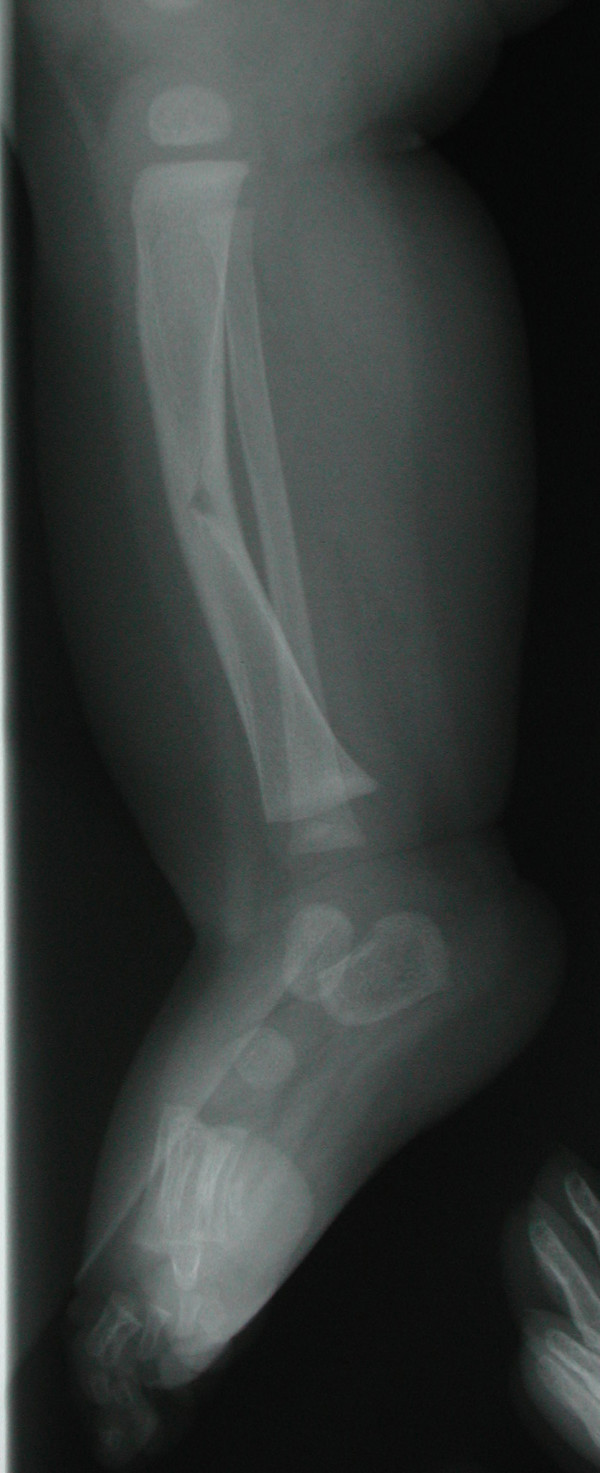
Greenstick type fracture of the mid-shaft of right tibia.

At 8 days of age, he was assessed by a pediatric orthopedic surgeon and the previous findings were confirmed. Magnetic resonance imaging (MRI) of the right leg was ordered. Congenital pseudarthrosis was in the differential diagnosis. This raised the possibility of NF1 and thus the patient was referred to the Pediatric Neurology and to the Medical Genetics services for evaluation. Radiographs at age 4 months identified a healing greenstick fracture of the right tibial mid-shaft and a straight fibula. Interpretation of the radiograph was complicated by the presence of a fracture and periosteal reaction such that tibial duplication could not be excluded [Figure 1]. There was partial duplication of the phalanges and first metatarsus, but given the patient's young age, no comment could be made on the medial cuneiform as it had not ossified. Radiographs taken subsequently when the child was older suggested possible duplication of the medial cuneiform [Figure 2]. He was assessed by the pediatric neurologist at age 7 months. There were no stigmata of NF1 present on examination and his family history was negative for any neurocutaneous disorders. His development appeared to be age-appropriate. An MRI of the brain and right lower extremity was performed. No abnormality of the brain was identified. Tapering and cortical thinning of the mid-aspect of the tibia was observed. The anterior cortex appeared continuous while callus formation along the posterior aspect of the mid-tibia was present. There was no definitive evidence of duplication of the tibial medullary canal nor was a pseudarthrosis identified. There was a supernumerary digit arising from the first cuneiform of the right foot.

At age one year, he underwent surgical excision of the supernumerary toe with syndactylization of the right great toe to the second toe. He was first seen in Medical Genetics at age 15 months. The right foot showed good correction of the deformity. There was no clinical evidence of tibial bowing on the right. His left index finger remained radially deviated. Again no stigmata of a neurocutaneous disorder were present. His development was otherwise normal and he had been ambulating since age 12 months while wearing a cast following surgery. Follow-up at age 2 years 5 months demonstrated no problems with ambulation or foot pain. The patient was wearing wider shoes to accommodate the mild varus deformity of the right hallux. There appeared to be a clinically insignificant leg length discrepancy with the right lower extremity being shorter than the left.

The family history was extensively reviewed. The patient's father had a maternal first cousin who was born with bilateral clubfeet. Otherwise there was no other history of any musculoskeletal findings. The patient also had a maternal first cousin with anomalies consistent with a congenital cytomegalovirus infection. The parents were non-consanguineous and of northern European ancestry. The mother was in her sixth month of gestation. A diagnosis of CATBP was advanced. The parents were counselled regarding recurrence risks and were quoted a figure of <1%.

## Discussion

Congenital bowing of the long bones can be a feature of skeletal dysplasias such as camptomelic dysplasia and of metabolic bone diseases [2,3]. In these cases, symmetric involvement is usual. Congenital unilateral bowing of the tibia is an uncommon orthopedic condition. It is classified according to the direction of the angulation: anterolateral, anteromedial and posteromedial [2,3]. Anterolateral tibial bowing is commonly but not exclusively associated with NF1 and presents with a high risk of pathologic fracture and pseudarthrosis [2,3].

NF1 is a very common neurocutaneous disorder that commonly presents with café-au-lait macules, neurofibromas and other clinical manifestations. The diagnosis is made in an individual when two or more diagnostic criteria are fulfilled [1]. Pseudarthrosis is an osseous lesion that is included amongst the seven diagnostic criteria for NF1 and may be a complication of anterolateral bowing of the tibia [1]. NF1 can also present with central nervous system (CNS) tumors which is why neuroimaging studies may be performed even though some recommend that neuroimaging should not be performed routinely [4].

In this report, a 15-month old boy presented with congenital anterolateral tibial bowing associated with hand and foot anomalies. There was hallux duplication ipsilateral to the tibial involvement. The hand anomaly consisted of radial deviation of the left index finger. The child was otherwise healthy and demonstrated age-appropriate development. Specifically, he did not have any café-au-lait macules or any other skin findings suggestive of a neurocutaneous disorder. Because of the association between anterolateral tibial bowing and NF1, a neuroimaging study of the brain was requested. This did not reveal any abnormalities. Subsequently, he was diagnosed with CATBP, a disorder with a good prognosis that is not known to present with any CNS malformations.

CATBP is an uncommon but well-delineated genetic condition that was reported by Adamsbaum et al. [1991] in five children [3]. Tibial duplication with two medullary canals surrounded by distinct cortices has been described [2,3,5-7]. The fibula is often straight without any apparent signs of involvement, but with time there may be problems with proximal fibular overgrowth [5]. The duplication in the foot may not be limited to the hallux, but may also involve the first metatarsus and tarsus [5]. Four of the five patients also demonstrated associated hand malformations including syndactyly, pre-axial polydactyly and clinodactyly [3]. Radial deviation of the index finger, as in this patient, has been reported as an associated hand malformation [5] [Table 1]. Based on the published reports, the longterm prognosis for this condition appears to be generally favorable with spontaneous resolution of the tibial deformity, however there may be ongoing difficulties with pain and foot deformity [2,3,5-7].

**Table 1:** Published cases of congenital anterolateral tibial bowing and polydactyly.

Case	Gender	Age	Lower Limb Involved	Hand Anomaly	Reference
1	M	13 y	Right	None	2
2	M	18 mo	Right	None	2
3	F	17 mo	Left	Syndactyly, bifid thumb	3
4	M	12 y	Right	Clinodactyly	3
5	M	10 y	Left	Clinodactyly	3
6	M	7 mo	Right	Clinodactyly, hypertrophy	3
7	M	6 y	Right	None	3
8	M	9 mo	Left	None	5
9	M	6 y	Left	Radial deviation of indices	5
10	F	5–6 y	?	None	6
11	F	5–6 y	?	None	6
12	M	5–6 y	?	None	6
13	M	17 mo	Left	None	7

In our patient, the anterolateral tibial bowing was complicated by the presence of a fracture and resultant periosteal reaction making it difficult to confirm or exclude tibial duplication on x-ray initially. However evidence of tibial duplication could not be identified subsequently on MRI. He also had other radiographic findings such as partial duplication of the hallux and first metatarsus as well as a minor hand anomaly which have been reported as associated features in this condition. He remains pain free and does not appear to have a clinically significant foot deformity. He is being followed closely for evidence of any orthopedic complications.

From a child care perspective, this is an important entity to recognize and distinguish from NF1. NF1 is a common neurocutaneous disorder that is inherited in an autosomal dominant fashion and demonstrates age-related penetrance and considerable clinical variability [1,4]. A diagnosis of NF1 has potential implications for both the parents and the offspring of the affected individual. Management guidelines for children and adults with NF1 have been published [4]. Routine neuroimaging studies in patients with NF1 are not recommended unless there are signs of neurologic involvement [4]. This patient was unnecessarily investigated for NF1 because of its association with congenital anterolateral bowing of the tibia.

The etiology for CATBP remains enigmatic. All published cases have been isolated and recurrence in sibships has not been reported [2,3,5-7]. None of the published reports mention any associated cytogenetic abnormalities. There are also no reported cases of parent to child transmission. This may suggest an intrauterine or developmental abnormality rather than a Mendelian disorder. However, with so few cases reported in the literature, this is purely speculative. Additional reports in the future, especially of any familial cases, may help to determine the genetic basis of this condition and to make this a more recognizable condition amongst pediatricians.

## Conclusion

CATBP is a rare but recognizable entity that presents with anterolateral tibial bowing. It is generally associated with a favorable prognosis. The diagnosis is made clinically based on physical and radiographic findings. It should not be confused with NF1 or other causes of anterolateral bowing of the tibia. An accurate diagnosis is important to prevent unnecessary investigations.

## List of Abbreviations

NF1 Neurofibromatosis type 1

CATBP Congenital anterolateral tibial bowing and polydactyly

MoM Multiples of the Median

MRI Magnetic resonance imaging

## Competing interests

The author(s) declare that they have no competing interests.

## Authors' contributions

EL examined and diagnosed the patient, reviewed the medical literature, drafted and revised the manuscript and gave final approval of the version to be published
